# A Study on the Effect of Temperature Variations on FPGA-Based Multi-Channel Time-to-Digital Converters

**DOI:** 10.3390/s23187672

**Published:** 2023-09-05

**Authors:** Awwad H. Alshehry, Saleh M. Alshahry, Abdullah K. Alhazmi, Vamsy P. Chodavarapu

**Affiliations:** Department of Electrical and Computer Engineering, University of Dayton, 300 College Park, Dayton, OH 45469, USA; alshehrya1@udayton.edu (A.H.A.); Alshahrys1@udayton.edu (S.M.A.); alhazmia3@udayton.edu (A.K.A.)

**Keywords:** field programmable gate arrays, FPGA, RMS resolution, tapped delay line, temperature variations, time to digital converter

## Abstract

We describe a study on the effect of temperature variations on multi-channel time-to-digital converters (TDCs). The objective is to study the impact of ambient thermal variations on the performance of field-programmable gate array (FPGA)-based tapped delay line (TDL) TDC systems while simultaneously meeting the requirements of high-precision time measurement, low-cost implementation, small size, and low power consumption. For our study, we chose two devices, Artix-7 and ProASIC3L, manufactured by Xilinx and Microsemi, respectively. The radiation-tolerant ProASIC3L device offers better stability in terms of thermal sensitivity and power consumption compared to the Artix-7. To assess the performance of the TDCs under varying thermal conditions, a laboratory thermal chamber was utilized to maintain ambient temperatures ranging from −75 to 80 °C. This analysis ensured a comprehensive evaluation of the TDCs’ performance across a wide operational range. By utilizing the Artix-7 and ProASIC3L devices, we achieved root mean square (RMS) resolution of 24.7 and 554.59 picoseconds, respectively. Total on-chip power of 0.968 W was achieved using Artix-7, while 1.997 mW of power consumption was achieved using the ProASIC3L device. We worked to determine the temperature sensitivity for both FPGA devices, which could help in the design and optimization of FPGA-based TDCs for many applications.

## 1. Introduction

Many applications that require high-precision time measurement such as spacecraft missions, medical diagnostics, materials spectroscopy, light detection and ranging (LiDAR), and high-energy nuclear physics, use time-to-digital converters (TDC) [1,2,3,4,5,6,7]. TDCs are used in applications where the precise time interval between two digital signals is required. Many commercially available TDCs offer timing resolution of less than 10 picoseconds for single and multi-channel versions [8,9,10]. TDCs are commonly implemented using an application-specific integrated circuit (ASIC) or a field-programmable gate array (FPGA) platform. While both ASICs and FPGAs have certain advantages and limitations, FPGAs are the preferred choice for multi-channel TDCs due to their advantages of shorter development time, flexibility, and cost-effectiveness, as shown in Table 1. Moreover, FPGA devices can be reprogrammed, which make FPGAs well-suited for use in harsh and dynamic environments, including space and critical civilian applications [11,12,13,14].

Various FPGA-based TDC architectures have been developed to date, including course counters, phased clocks, tapped delay lines (TDLs), differentials, and pulse shrinking. For these uses, TDL architecture offers simplicity, reliability, low latency, and low resource utilization [13,15]. The internal resources of the FPGA device are utilized as delay elements to build the carry chains in the TDL. However, TDL architectures are susceptible to process, operating voltage, and temperature (PVT) variations [8,16,17,18], which deteriorate the TDC performance by changing both the uniformity and the propagation delay time of the internal logic resources of the FPGA.

The structure of the TDL TDC used in this work is represented by the block diagram shown in Section 2. The goal of using the TDL structure is to sample the propagation of the start signal through the delay lines using the system clock signal, which is assigned as the stop signal [19]. There are different schemes for implementing the TDL, including delaying the start signal, clock signal, or both through the delay elements. In this work, the start signal is delayed through a series of delay elements. The delay elements are chosen based on the available logic resources in the selected FPGA chip to build the TDL [13,14,15]. Won et al. [20] proposed two TDC structures based on TDL architecture to help solve both the non-linearity and clock skew issues. Their group analyzed the properties of clock skew on TDL TDC through numerical analysis (density test code) and the TDC transfer function using the Xilinx Virtex-6 (ML605) device. Favi and Charbon [21] explained that turbo mode is presented to enable sub-nanosecond time resolution of the conversion rate, up to 300 M sample/s, and to use a range higher than 50 ns. Song and Liu [22] presented an FPGA-based TDC where the FPGA’s committed carry lines were used as delay elements. They obtained a time measurement resolution of 50 ps after calibration.

This study shows that the new generation of FPGA devices can provide stable and high performance in the presence of large temperature variations using Artix-7 and ProASIC3L. The radiation-tolerant ProASIC3L device built for space applications maintains better stability in terms of thermal sensitivity and power consumption compared to the Artix-7. An overview of some previous studies covering the impact of thermal variations on TDC performance is listed in Table 2. The proposed designs are tested under ambient temperatures ranging from −75 to 80 °C to study the effect of temperature variations. Compared to the temperature range considered in our work, the minimum temperature analyzed was −21 °C by [23], and the maximum temperature was 85 °C by using Xilinx Virtex-4 and Artix-7 devices [24,25]. We achieved a stable RMS resolution of 24.7 and 554.59 picoseconds using Artix-7 and ProASIC3L, respectively. Moreover, we achieved low power consumption of 0.968 W and 1.997 mW for Artix-7 and ProASIC3L, respectively. We determined the temperature sensitivity factors for both FPGA-based TDC systems. The proposed TDCs in this study are suitable for use in various safety-critical, mission-critical, telecommunications, space, consumer, medical imaging, and industrial applications. Table 2 and Section 5 justify the achieved results of the proposed TDCs with other methods in more detail.

This article is organized as follows. Section 2 describes the methodology employed, data collection methods, analysis techniques, the specifications of the FPGA devices, the performance impacts due to ambient thermal variations, and the TDC architecture used for the proposed TDCs based on the TDL approach. The implementation details and utilization statistics for the FPGA-based TDCs are presented in Section 3. The algorithms employed for time measurements are detailed in Section 4. The results and discussions, including the statistical, thermal variation, and power consumption analyses, are presented in Section 5. The conclusions are given in Section 6.

## 2. Methodology

FPGA-based TDCs are used in various civilian, industrial, and space applications that are required to operate under a wide range of operational temperatures [31,35,37,38]. The proposed TDCs have multi-channel capability, up to 64 and 16 channels using Xilinx Artix-7 and Microsemi ProASIC3L devices, respectively. The internal resources used to design the TDC architectures are sensitive to (PVT) variations, which impact the TDC performance by changing the intrinsic delay time of the FPGA’s internal logic resources.

- Process variations: Continued development of FPGAs has enabled faster transistor switching rates and less power consumption, yet for high-volume production, there are variations during semiconductor manufacturing that lead to changes in the FPGA’s performance [20,39].
- Voltage variations: The core voltage for most modern FPGAs is within the range of 1.2–1.5 volts [39,40,41,42,43]. Although the voltage threshold can be regulated using the built-in voltage regulators on the FPGA devices, in some conditions, a small variation of a few millivolts can impact the TDC accuracy.
- Temperature variations: The ambient temperature and device operational temperature vary over time, which affects the FPGA logic fabric performance [39,44,45]; therefore, it is crucial to study the impact of temperature variation on the performance of FPGA-based TDCs.

**Figure 1:**
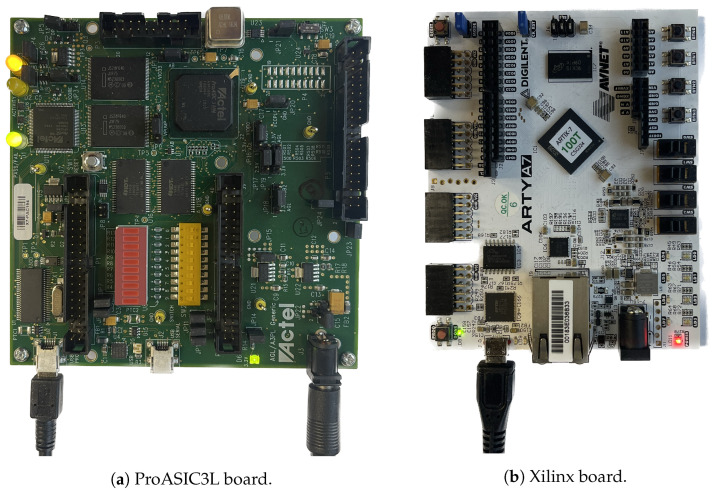
Photographs of the FPGA boards utilized.

In this study, we focused on analyzing the impact of thermal variations on the performance of FPGA-based multi-channel TDL TDCs under temperature variations ranging from −75 to 80 °C. Multichannel TDCs are designed and implemented on two different FPGA platforms, Xilinx and ProASIC3L, as shown in Figure 1. We tested the TDCs under different temperature values with both rising and falling ambient temperatures. During our testing, we measured the performance metrics, including time resolution RMS values versus temperature, under short-term (one hour with 10 °C increments) and long-term (12 h at a fixed temperature of −10 °C) conditions. For both conditions, we analyzed the impact of temperature variations on the TDC performance to study the design stability. Finally, we examined and compared the collected data by observing the time RMS resolution measurements of the TDC system obtained under the two test conditions.

### 2.1. Specifications of Selected FPGAs

Xilinx Artix-7 and Microsemi ProASIC3L FPGAs have been used to implement a wide range of complex digital systems. Some of their technical details are covered in Table 3. While TDL logic structures are available in both Xilinx Artix-7 and ProASIC3L FPGAs, their available logic resources are different. The primitive CARRY4 logic block available in Artix-7 has four internal multiplexers and is used to build the TDL. The AND2 gate in the ProASIC3L FPGA is used as the delay element to build the TDL. In addition, the propagation time differs from one FPGA device to another. The propagation time of a single multiplexer in the CARRY4 logic cell is about 25 ps, while it is expected to be in the range of 480 ps to 1050 ps using the available resources in ProASIC3L [39,40]. There are many logic gates available in the ProASIC3L FPGA that have almost the same propagation delay time, such as AND2, NAND2, OR2, and NOR2. In the proposed design implemented on ProASIC3L, the AND2 gate is selected as the logic gate to build the carry chains of the TDL, as it is one of the fastest gates in the device, with an expected propagation time of 570 ps for every single delay element. More specifications of the FPGA devices are illustrated in Table 3.

### 2.2. The Proposed TDL TDC

TDL TDCs often rely on simple architectures and require low FPGA resource utilization [11]. The TDL encodes the time difference between the digital instants into several bits in thermometer time code using the propagation delay time through a series of internal logic blocks in the FPGA [13,15]. This thermometer time code is then converted into a number using a binary encoder. The encoder output reflects how many delay elements have passed by the START signal at the positive edge of the STOP signal.

### 2.3. Tapped Delay Line (TDL) on Xilinx and ProASIC3L FPGA Boards

**Figure 2:**
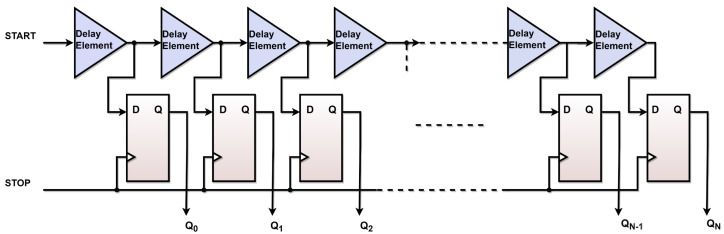
Architecture of the proposed tapped delay line in a single channel TDL TDC.

The purpose of utilizing the TDL is to convert the time intervals between the start and the stop pulses into a binary representation called a time thermometer time code [$Q_{0}$$Q_{1}$, …, $Q_{N}$] using the propagation time of the internal logic resources. The architecture of the proposed design in a single-channel TDL TDC for both FPGA boards is shown in Figure 2.

### 2.4. Xilinx FPGA-Based TDL TDC

**Figure 3:**
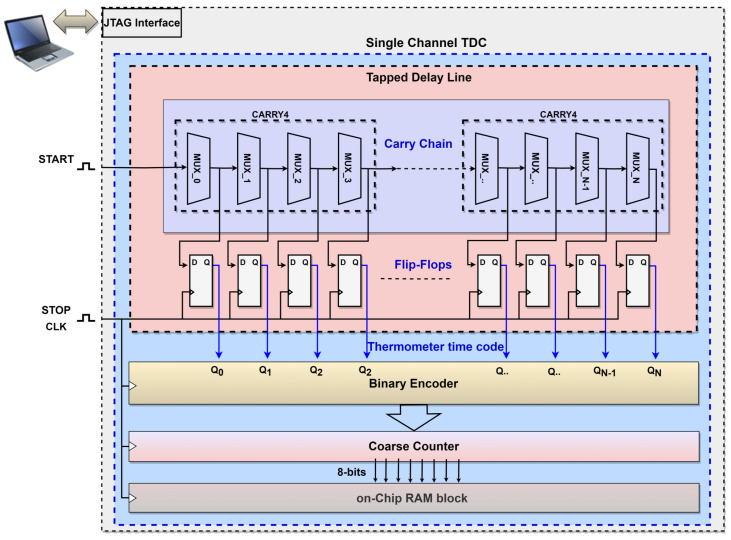
Block diagram of a single-channel TDC using TDL architecture on the Xilinx Artix-7.

Xilinx FPGAs provide the CARRY4 primitive, a fast-carry logic with look-ahead that can be utilized as a delay element to build the TDL unit [40]. The architecture of the TDL unit on the Xilinx FPGA device is constructed by cascading a series of 64 CARRY4 followed by a flip-flop array. Since each CARRY4 has four internal multiplexers, the total number of TDL output bits is 256. The START signal is connected to the carry initiate input (CINIT) pin of the first CARRY4 structure to initiate the time measurement. The CARRY4 blocks have four independent delayed bits, carry output pins CO[0] to CO[3]. The last output bit, CO[3], is cascaded to the next CARRY4 block through its carry input (CI) pin. This process continues for the rest of the 64 CARRY4 to build a 256-bit delay line for a single-channel TDC. Every delay line consists of one multiplexer followed by two flip-flops. The STOP signal serves as a sampling signal by being commonly connected to the clock (CLK) input of the flip-flop arrays. At the rising edge of the STOP signal, the thermometer time code is generated by reflecting the logical state of the flip-flop’s arrays at the output of the TDL unit. Therefore, the total number of ones represents the time intervals in the thermometer time code. Subsequently, the encoder counts the total number of taps by identifying the last transition bit in the thermometer code. This enables estimation of the delay time between the two events using the known periodic time of the STOP signal, also defined as the sampling frequency. The block diagram of a single-channel TDC using TDL architecture on the Xilinx Artix-7 is shown in Figure 3.

### 2.5. ProASIC3L FPGA-Based TDL TDC

**Figure 4:**
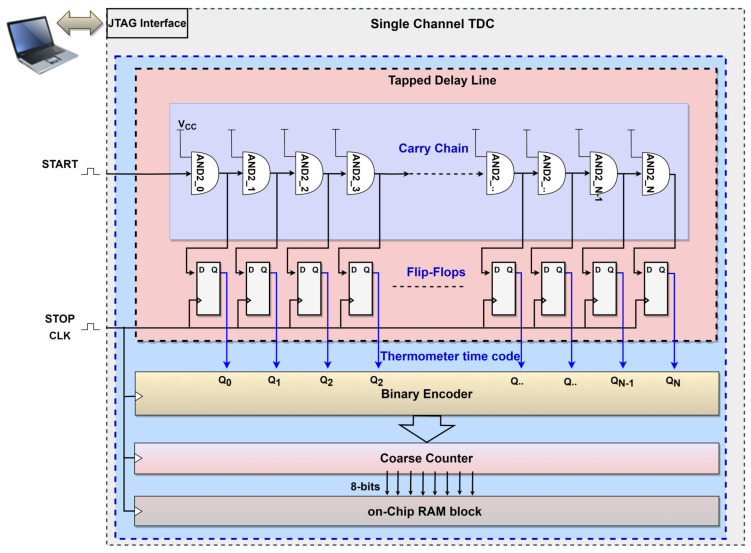
Block diagram of a single-channel TDC using TDL architecture on the ProASIC3L.

The ProASIC3L offers the AND2 logic block to build the TDL. The START signal is connected to the first input pin of the AND2 gate, while the second input is commonly connected to the VCC of the chip. This configuration occupies the AND2 gate as a single delay element. The output pin of the first AND2 gate is connected to the input of the next AND2 gate and then to the input of the flip-flop arrays to build a 256-delay line. Simultaneously, the STOP signal is used as a sampling signal by connecting it to the clock (CLK) input of the flip-flop arrays. Every AND2 output pin has an independent delayed bit from $Q_{0}$–$Q_{N},\;N=255$, to generate thermometer time codes of 256 bits to track the START signal propagation in the TDL carry chain. Then, the thermometer time code values are used to reflect the time intervals between the START and STOP signals. Subsequently, the encoder counts the total number of taps by identifying the last transition bit in the thermometer code. This enables estimation of the delay time between the two events using the known periodic time of the STOP signal, also defined as the sampling frequency. Figure 4 illustrates the block diagram of a single-channel TDC using TDL architecture on the ProASIC3L.

### 2.6. Encoder

An 8-bit binary folded thermometer-to-binary encoder is used in both FPGA devices due to its high precision and low resource consumption. Typically, the measurement accuracy of the TDC mostly relies on the precise identification of the START transition in the 256-bit output of the TDL. This transition indicates the exact number of delay taps and is important for obtaining accurate time measurements. The START signal propagates in a sequential pattern; therefore, bubble suppression capability is required in the employed encoder. Bubble errors occur as a result of spurious transitions in the thermometer time codes, which can adversely affect the time measurement accuracy. In addition, some of the uneven propagation delays, the skew in the sampling clock, and the meta-stability of the flip-flop arrays could generate a single or various inverted bits (0 value) between 1 s, e.g., [11110111010000 …]. Therefore, it is essential to resolve such errors to improve the precision of the TDC. The encoding process begins by dividing the thermometer time code in half to detect the 1–0 transition bit as follows. The thermometer time code is divided into $Q_{0}$–$Q_{127}$ and $Q_{128}$–$Q_{255}$. If the transition bit at the center equals zero, the upper half of the thermometer time code will be discarded, and the lower half is then processed. The same process is repeatedly applied to the rest of the code until the transition bit is determined. The encoder output is an 8-bit binary number for each time measurement event stored in the on-chip BRAM using the JTAG interface block design.

## 3. Time Measurement Algorithms

Both the FPGAs have built-in crystal oscillators that are used to generate a specific frequency to drive the required signals for the TDCs, such as the reference clock and the STOP and START signals. The signals with different frequencies are generated by a mixed-mode clock manager (MMCM) available in the IDE software tools, described in Section 4. During the measurement process, the signals are sent to the TDC to generate random time shifts or time intervals (TIs). The number *N* in Equation (1) represents the TDC output data, or the total number of the delay elements passed by the START signal as described previously:

(1) $$N=\frac{\Delta T}{T_{LSB}}$$

where Δ*T* is the time interval between the START and STOP signals. Assuming the time delay of all the TDL bins is distributed evenly within one clock period, the time interval between START and STOP signals can be define as Δ*T* using Equation (2):

(2) $$\Delta T=N*T_{LSB}+\varepsilon$$

The delay time of a single delay element in the delay-line $T_{LSB}$ is the time of least-significant-bit. The value of the LSB in the TDC is determined by the averaged bin size using Equation (3):

(3) $$T_{LSB}=\frac{\Delta T}{N}+\varepsilon$$

where ε is the quantization error that arises from reflecting the incorrect status of the flip-flops, in which it causes a bubble error in the TDC outputs. Using the Tool Command Language script, two signals with different frequencies are used to run the design on the FPGA board for analyzing and characterizing the TDC performance, depending on the time measurements. The first signal, STOP, is set as a reference clock to drive the system and sample the TDC results. In contrast, the next signal is set to be the START signal to initiate the measurement process. This frequency difference will create random time shifts between the two events, START and STOP signals. Accumulating the TDC outputs helps build the histogram and store the data in the internal BRAM of the FPGA. In this process, the total number of the registered events is 50,000, which is transferred to a computer through the JTAG interface in text format at the end of the test process. The final step is to determine the statistical charts’ results and the TDC performance’s precision using a custom Python script.

### Averaging Process

Due to the uneven bin width of the delay times caused by PVT variations, an averaging process is used to find the time for the least square bit of the proposed TDC design. The output data contain the number of bits, *N*, that are collected from sending the random time shifts between START and STOP signals to the TDC. All of the activated bin numbers are stored in the BRAM and then transferred to the computer to be processed using the Python script. Since some bins have more counts than others, the average time is calculated using an averaging process by extracting the bin numbers from the raw output data using Equation (5). The calibration of the estimated least square bit is based on the known periodic time of the STOP signal.

**Figure 5:**
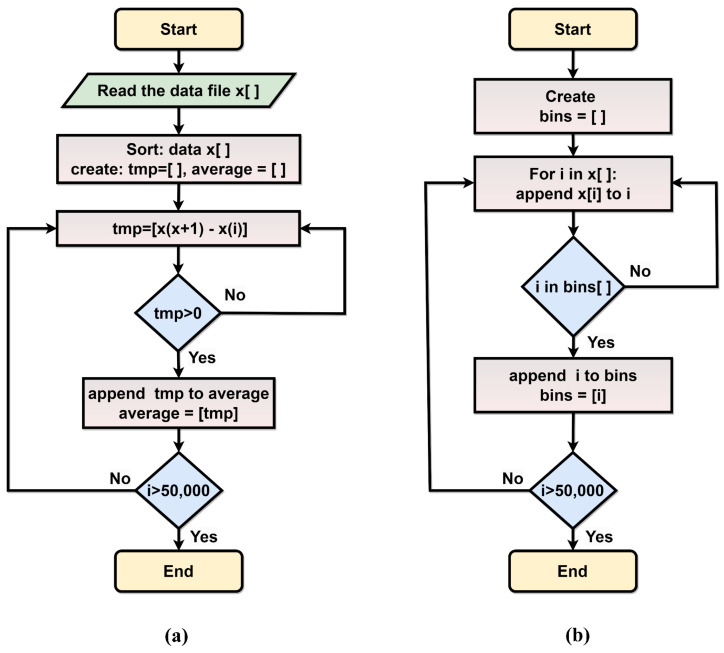
Flowchart of (**a**) Averaging process (**b**) Finding the active bins.

The flow charts in Figure 5 illustrate the sequencing process applied to the TDC data to determine the average precision for the single delay element. Within one clock period of the sampling frequency $T_{C},$ the delay time for the single delay element is estimated using Equation (4):

(4) $$T_{LSB}=\frac{T_{C}}{N}+\varepsilon .$$

The average bin width $T_{avg}$ is determined according to the activated bins avg(n) throughout the measurement results. Then, the collected bin numbers are sorted from the smallest bin number to find the bins that successfully registered the time intervals. By applying Equation (5), the average bin width for the 256-bit tapped delay line (TDL) can be calculated:

(5) $$T_{avg}=\frac{\sum_{i=n}^{N}avg\left(n\right)}{N}*T_{LSB}.$$

## 4. Implementation

The implementations of the TDC on both devices are obtained using two different integrated development environments (IDEs). Xilinx Vivado v2021.1 software is used to program the Artix-7 FPGA, and Libero SoC v11.9 SP6 is used to program the ProASIC3L, both through the JTAG interface.

### 4.1. Implementation on Xilinx FPGA

**Figure 6:**
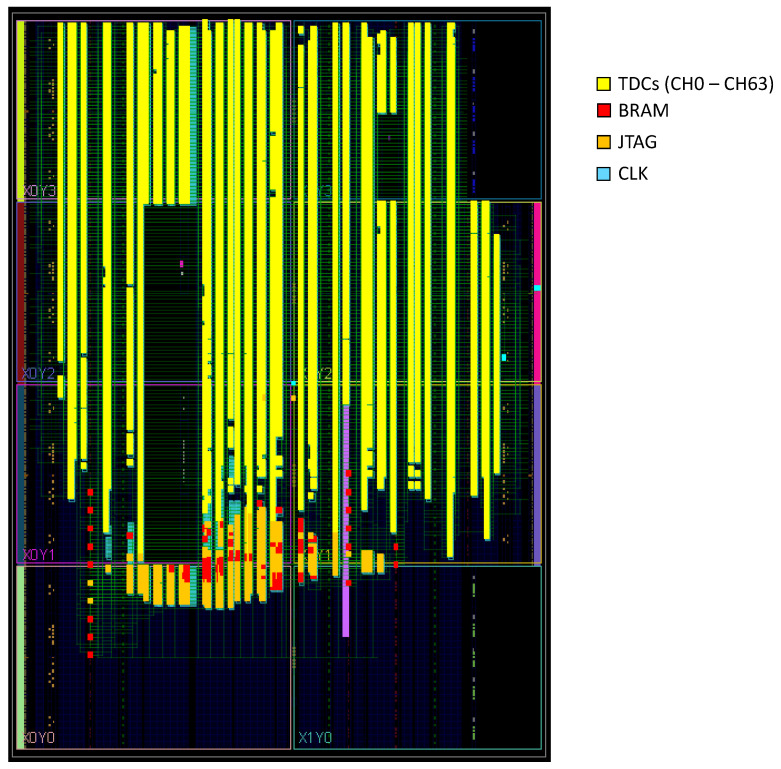
64-channel TDL TDC implementation.

The pre-built peripheral blocks used to design the TDC include the clock wizard, IP inter-connector, and BRAM controller, as shown in Figure 6. The Xilinx floor planning tool is used to assign the required logic resources to implement the TDC system. There are eight clock regions available within the Artix-7 device, in which we placed the TDC system, as presented in Figure 6. All 64 channels of the TDC are created inside most of the available clock regions. The green lines indicate the routing between the internal resources to build the design. The proposed design includes the TDL, the encoder, and the rest of the logic resources required for the JTAG interface blocks. Table 4 shows on-chip resource utilization statistics to implement the proposed 64-channel TDC. It also visualizes the utilization in percentages for the lookup table (LUT), lookup table of RAM (LUTRAM), flip-flops, block RAM configurable memory module (BRAM), and mixed-mode clock manager (MMCM) resources.

### 4.2. Implementation of TDC on ProASIC3L

**Figure 7:**
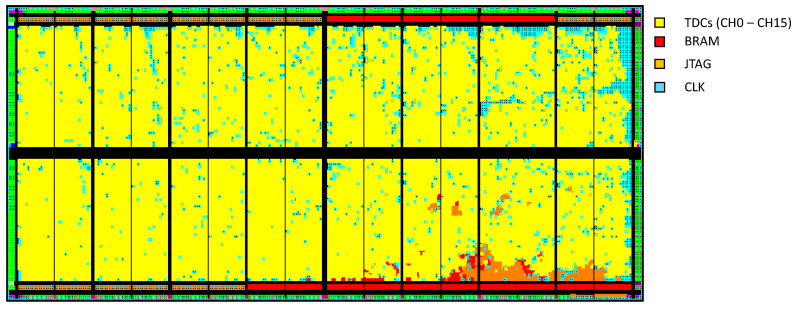
16-channel TDL TDC implementation on the ProASIC3L device.

Various pre-built peripheral blocks used to design the TDC include the clock wizard, IP interconnector, and BRAM controller, as shown in Figure 7. Table 5 shows on-chip resource utilization statistics required to implement the proposed 16-channel TDC. The multiview navigator tool is used to view the implemented logic resources of the TDC. There are 32 clock regions available in the FPGA chip, in which we placed the TDC system, as presented in Figure 7.

## 5. Results and Discussion

The performance of our TDL TDC designs is evaluated for both devices in terms of statistical, thermal variations, and power analysis. Statistical analysis covers the measurement accuracy, interpolation linearity, the activated bins, as well as the bin number count versus bin width in the picosecond range. The measurement results of the multi-channel TDCs are presented by evaluating their performance and precision in two scenarios, including averaged precision and root mean square (RMS) resolution. The thermal analysis includes the short- and long-term thermal variation discussions and findings. Finally, the power consumption analysis is covered for the proposed multi-channel time-to-digital converters.

### 5.1. Statistical Analysis of the Proposed TDL TDC on Both FPGAs

**Figure 8:**
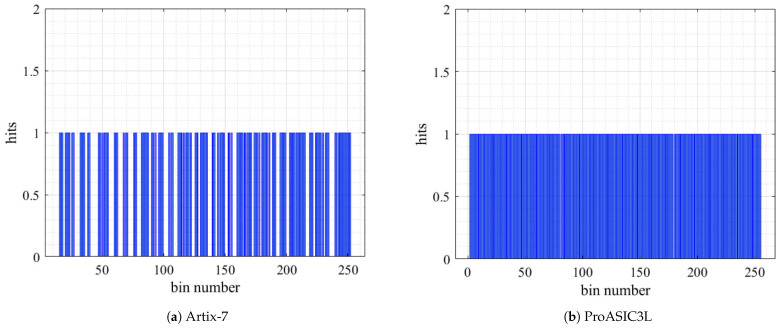
Number of hits versus bin number.

The performance of the TDCs is shown in Figure 8a,b. The charts illustrate the number of activated bins in the delay lines during the measurement process within the delay elements in the Artix-7 and ProASIC3L devices. The activated bins indicate the bins that successfully detected the time intervals.

**Figure 9:**
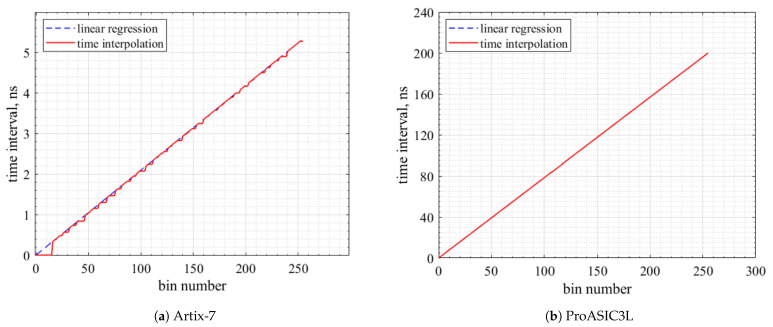
Time interpolation linearity.

Figure 9a,b depicts the multi-channel TDC time interpolation linearity for both FPGA devices. The red line in the charts shows some bins along the tapped delay lines (TDLs) that did not detect the time intervals; these bins are represented by the small triangles underneath the ideal case line in blue (linear regression). The ProASIC3L provided better linearity compared to the Artix-7.

**Figure 10:**
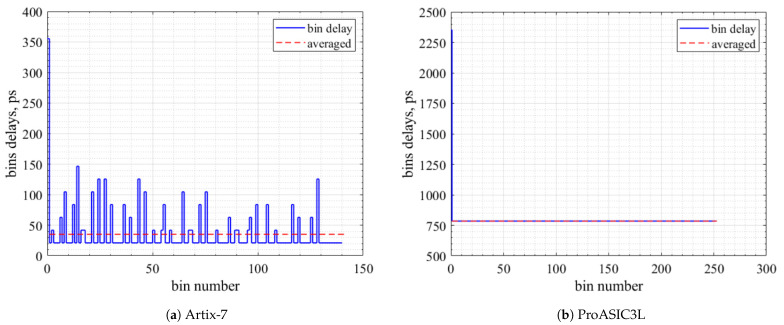
Delay time versus bin number.

The horizontal line in Figure 10 indicates the average precision, and a few bins, as shown, reflect the ultra-wide bin widths in the multi-channel TDC performance. Table 6 shows the achieved average precision and the RMS resolution of the proposed TDCs.

**Figure 11:**
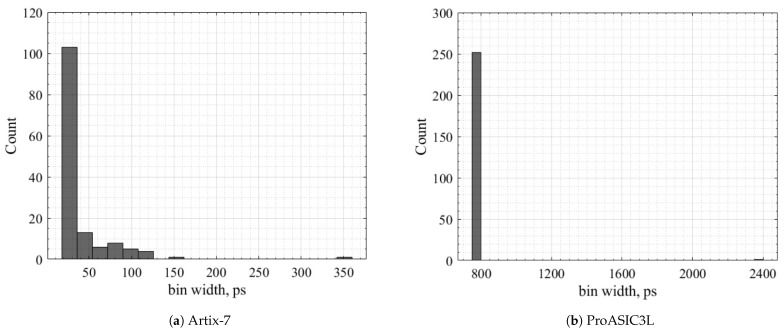
Bin width distribution.

The histogram bars in Figure 11 show the counts of the bins over the bin width in picoseconds.

### 5.2. Thermal Variation Analysis

The temperature sensitivity of the FPGA-based TDC performance is studied by changing the ambient temperature on the FPGA board using a thermal chamber (Thermotron model SE-300). This equipment is a laboratory thermal chamber capable of maintaining a wide range of selected temperatures to conduct thermal variation tests as required. First, we placed the FPGA boards at a fixed temperature for one hour at each temperature in the selected range from −75 to 80 °C to ensure a stable ambient temperature. Then, we ran the TDC and observed the results in order to study the time measurement drift at each given temperature. Then, for the long-term test, we fixed the ambient temperature and ran the TDC for a duration of 12 h.

**Figure 12:**
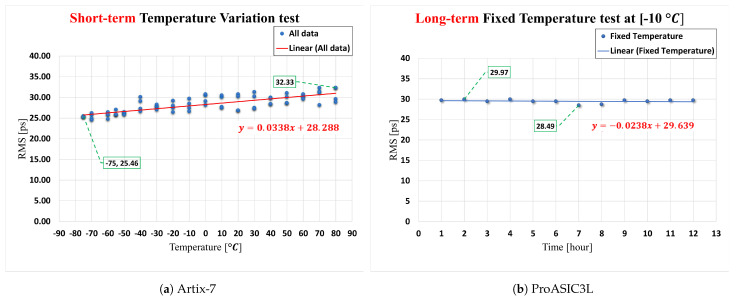
The influence of short/long-term temperature variations on TDC performance.

For the Xilinx Artix-7 FPGA-based TDC board, the results demonstrate the effect of temperature fluctuations within the range −75 to 80 °C for the short-term test, as shown in Figure 12a, while the results of the long-term test are shown in Figure 12b. The determined temperature sensitivity factor for the short-term variation test is 0.0338 ps/°C, as illustrated in Figure 12a. For the long-term test, the temperature sensitivity factor is −0.0238 ps/°C, as shown in Figure 12b.

**Figure 13:**
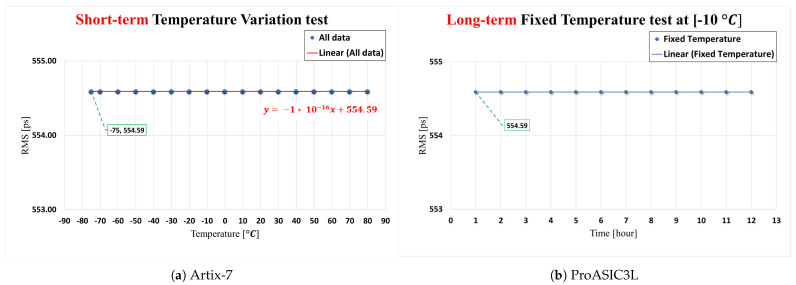
The influence of short/long-term temperature variations on TDC performance.

For the FPGA-based TDC implemented on ProASIC3L, the results demonstrate the effect of temperature fluctuations within the range −75 to 80 °C for the short-term test, as shown in Figure 13a, while the results of the long-term test are demonstrated in Figure 13b. The TDC’s performance on the ProASIC3L FPGA device demonstrates a consistent and stable output even when subjected to both short- and long-term thermal variations.

### 5.3. Power Consumption Analysis

**Figure 14:**
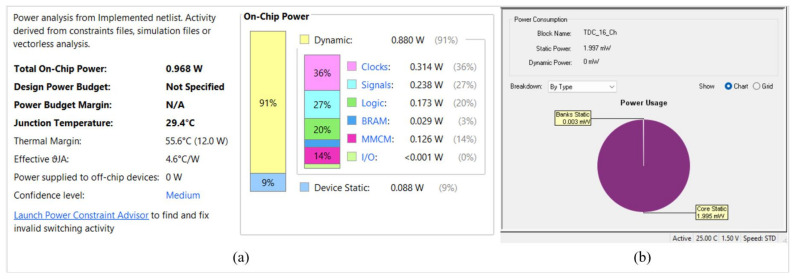
Power consumption of (**a**) Xilinx Artix-7 and (**b**) ProASIC3L.

The power analysis for our multi-channel TDCs is shown in Figure 14a,b. The power supply voltage is 1.25 V on the Artix-7 core of the Xilinx, compared to 1.5 V on the ProASIC3L core of the Microsemi. Stable power consumption was observed for both FPGAs. Total on-chip power consumption of 0.968 W was achieved by the proposed design in Artix-7, while 1.997 mW was achieved by ProASIC3L.

The presented results from the statistical, thermal variations, and power consumption analyses suggest that the multi-channel time-to-digital converters (TDCs) proposed for use on the Artix-7 and ProASIC3L platforms possess the ability to sustain consistent performance throughout a wide range of temperature variations. While the ProASIC3L device exhibits insensitivity to temperature fluctuations, the Artix-7 device demonstrates a significantly low temperature sensitive factor of 0.0338 ps/°C. This allows us to achieve a root mean square (RMS) resolution of 554.59 ps using the ProASIC3L and, for the Artix-7, a root mean square (RMS) resolution ranging from 24.75 to 32.33 ps within the specified temperature range. The TDC performance achieved using the two FPGAs can be justified by other methods in Table 2.

## 6. Conclusions

We studied the effect of thermal variations on multi-channel TDL TDC architectures, while simultaneously meeting the requirements of high-precision time measurement, low-cost implementation, small size, and low power consumption. This study demonstrates that the new generations of FPGA platforms can maintain stable and high performance even in the presence of large temperature variations. For our implementation, two FPGA devices were employed, Artix-7 and ProASIC3L. The radiation-tolerant ProASIC3L device built for space applications offers better stability in terms of thermal sensitivity and power consumption compared to the Artix-7. We employed a laboratory thermal chamber to ensure precise ambient temperature control within the desired range of −75 to 80 °C to study the effect of temperature variation on the TDCs. By utilizing the Artix-7 and ProASIC3L devices, we achieved RMS resolution of 24.7 and 554.59 picoseconds, respectively. We achieved low power consumption of 0.968 W and 1.997 mW for Artix-7 and ProASIC3L, respectively. We determined the temperature sensitivity factors for both FPGA-based TDC systems suitable for use in various safety-critical, medical imaging, mission-critical, space, telecommunications, consumer, and industrial applications.

## Figures and Tables

**Table 1:** Advantages and disadvantages of FPGA- and ASIC-based TDCs.

Platform Type	Pros	Cons
FPGA	Re-configuration of the TDCs without requiring hardware changes.	Require higher power consumption.
	High performance using high-speed digital signal processing techniques.	
	Easier to customize the TDCs to meet specific application requirements.	
	Lower cost, particularly if fewer FPGA resources are required to implement the TDCs.	
	Shorter development time.	
ASIC	Fully customized for optimum performance of the TDC for a specific application.	Lack of flexibility; ASICs cannot be reconfigured or modified once they are fabricated.
	Can be designed to minimize power consumption.	ASIC development is more expensive in low-volume production.
		ASIC designs are time-consuming processes, particularly if the designs are large or complex.

**Table 2:** Overview of studies.

Ref	Method	Device	RMS [ps]	Precision [ps]	Dead Time [ns]	Temperature Correction	Temperature Range [°C]	Temperature Sensitivity [ps/°C]	Power Consumption [W]
[26]	DL ^1^	ProASIC2	100	70	NA ^2^	NA	[−20–60]	0.5	0.260
[22]	LSPM ^3^	Kintex-7	1.29	3.54	NA	NA	[10–30]	NA	0.453
[27]	Counter-interpolator	Virtex-4	25	50	10	Automatic	[31–61]	0.047	NA
[28]	Counter-interpolator	A3PE1500	150	440	25	Automatic	[25–50]	0.6	1
[29]	VDL	A3PE1500	42	16.4	200	Automatic	[−5–55]	NA	NA
[23]	Counter-interpolator	A3PE1500	127	427	25	Automatic	[−21–71]	0.56	0.050
[30]	TDL	Kintex-7	85.7	NA	30	Automatic	[35–75]	0.5	NA
[31]	TDL WU ^4^	Cyclone II	21.8	30.9	NA	Automatic	[10–70]	NA	NA
[32]	TDL	Artix-7	56	156	NA	NA	NA	NA	43
[20]	TDL	Virtex-6	10	12.8	20	Real-Time	[10–50]	NA	NA
[24]	TDL	Virtex-4	120	NA	7	NA	[45–85]	6	NA
[25]	TDL	Artix-7	15	28	10	NA	[25–85]	NA	NA
[33]	Merged DL	Kintex-7	4.3	NA	50	NA	[40–70]	0.64	NA
[34]	TDL	APA1000	550	180	6400	NA	NA	NA	NA
[35]	NUMP ^5^	Cyclone 10	8.8	NA	NA	Automatic	[5–80]	0.054	0.039
[36]	NUMMP ^6^	Kintex-7	1.87	2.79	8	Re-TSM ^7^	[20–60]	NA	0.740
[37]	MCS ^8^	Kintex-7	1.3	4.6	8	Self-adaptation	[25–70]	0.0002	0.563
This work	TDL	Artix-7	24.7	35	10	Automatic	[−75–80]	0.0338	0.968
	TDL	APA1000	554.59	784.31	1	Automatic	[−75–80]	0	<0.002

^1^ DL: Delay Line. ^2^ NA: Not Available. ^3^ LSPM: Large-Scale Multiphase Matrix. ^4^ TDL WU: Tapped Delay Line Wave Union. ^5^ NUMP: Nonuniform Monotonic Phase. ^6^ NUMMP: Nonuniform Monotonic Multi Phase. ^7^ Re-TSM: Remarking the Time Scales Method. ^8^ MCS: Multichain Cross Segmentation.

**Table 3:** Specifications of the FPGAs used in our work.

Specifications	ProASIC3L	Xilinx Artix-7
FPGA Part Number	M1A3P1000L-FGG484	XC7A100TCSG324-1
Oscillator for system CLK	Yes	Yes
Powered using USB cable	Yes	Yes
Logic cells	1,000,000	101,440
Memory devices	4 MB of SRAM	4860 kbits
	16 MB of flash memory	
Supported by	Libero SoC ^1^ v11.9 SP6	Xilinx’s Vivado v2021.1
Radiation-tolerant technology	Yes	No
Power	Ultra-Low	Low
On-chip XADC ^2^	NA	Yes

^1^ SoC: system on chip. ^2^ XADC: analog-to-digital converter.

**Table 4:** FPGA resource utilization on the Artix-7 FPGA.

Resource	Utilization	Available	Utilization %
LUT ^1^	11,909	63,400	18.78
LUTRAM ^2^	410	19,000	2.16
FF ^3^	54,114	126,800	42.68
BRAM ^4^	18.5	13.70	13.7
MMCM ^5^	1	6	16.67

^1^ Lookup Table. ^2^ Lookup Table of RAM. ^3^ Flip-Flops. ^4^ Block RAM. ^5^ Mixed-Mode Clock Manager.

**Table 5:** FPGA resource utilization on ProASIC3L.

Resources	Utilization	Available	Utilization %
CORE	22,588	24,576	91.91
IO (w/clocks)	1	300	0.33
Global (Chip + Quadrant)	6	18	33.33
PLL ^1^	1	1	100
RAM	16	32	50

^1^ Phase-Locked Loop.

**Table 6:** The achieved results of the proposed TDCs using Artix-7 and ProASIC3L devices.

TDL TDC	Artix-7	ProASIC3L
Average Precision (ps)	35	784.31
RMS Resolution (ps)	24.75	554.59

## Data Availability

The data presented in this study are available upon request from the author A.H.A.
